# Predictive value of *STMN1* gene promoter polymorphism (−2166T>C) in patients with advanced NSCLC treated with the combination of platinum compounds and vinorelbine

**DOI:** 10.1007/s00280-015-2831-7

**Published:** 2015-07-29

**Authors:** Radosław Mlak, Paweł Krawczyk, Marzanna Ciesielka, Iwona Homa, Tomasz Powrózek, Monika Prendecka, Piotr Kozioł, Janusz Milanowski, Teresa Małecka-Massalska

**Affiliations:** Department of Human Physiology, Medical University of Lublin, Radziwiłłowska 11, 20-080 Lublin, Poland; Department of Pneumology, Oncology and Allergology, Medical University of Lublin, Jaczewskiego 8, 20-954 Lublin, Poland; Department of Forensic Medicine, Medical University of Lublin, Ceramiczna 1, 20-150 Lublin, Poland

**Keywords:** Non-small cell lung cancer, Polymorphism, *STMN1*, Chemotherapy

## Abstract

**Purpose:**

The combination of platinum compounds and vinorelbine is often used as a first-line treatment in patients with locally advanced or metastatic non-small cell lung cancer (NSCLC), without activating *EGFR* mutations and *ALK* rearrangement. Unfortunately, less than half of the patients benefit from chemotherapy. Moreover, majority of patients are exposed to a number of side effects of chemotherapy. Stathmin-1 (STMN1, oncoprotein 18) affects significantly microtubule dynamics and formation of the mitotic spindle. Therefore, the change in the *STMN1* gene may be a potential predictive factor of response to treatment regimens containing a cytostatics-disrupting microtubule dynamics (vinca alkaloids and taxoids). The aim of the study was to determine the relationship between a single nucleotide polymorphism (SNP) of the promoter of *STMN1* gene −2166T>C) and the effectiveness of chemotherapy based on platinum compounds and vinorelbine in patients with NSCLC.

**Methods:**

The investigated population consisted of 110 locally advanced or metastatic NSCLC patients treated with first-line chemotherapy, based on platinum compounds and vinorelbine. SNP was determined by SNaPshot™ PCR in DNA isolated from peripheral blood leukocytes.

**Results:**

The median progression-free survival (PFS) was significantly shorter in carriers of TT genotype of the *STMN1* gene compared with patients with CC or CT genotypes (2.75 vs. 6.5 months; *p* = 0.0033; HR 5.91, 95 % CI 1.81–19.33). Evaluated SNP did not significantly affect the response to treatment and the overall survival of the patients.

**Conclusion:**

Rare TT genotype of *STMN1* gene may be an unfavorable predictive factor of chemotherapy based on platinum compounds and vinorelbine, in patients with NSCLC.

## Introduction

Lung cancer still remains the most common malignant neoplasm in developed countries. In 2012, 1.8 million new cases and 1.59 million deaths due to lung cancer were recorded. Most prevalent histological subtype (85 % of cases) of this cancer is non-small cell lung cancer (NSCLC) [1]. The majority of patients with NSCLC are diagnosed at an advanced stages of the disease (inoperable cases IIIA, IIIB, IV), which also disqualifies them from radical surgery. These patients can only be subjected to chemotherapy (CTH) or radiochemotherapy (RCTH) of moderate efficacy.

Currently, standard first-line treatment of patients with locally advanced and metastatic NSCLC based on a combination of platinum compounds with third-generation drug (gemcitabine, vinorelbine, docetaxel, and pemetrexed). Unfortunately, objective response rate (ORR) obtained on the basis of this kind of treatment usually does not exceed 30–40 %. Use of first-line CTH extends median overall survival (OS) by approximately 1.5 months and increases the probability of one-year survival by approximately 9 % compared with patients treated with best supportive care [2].

At present the highest efficiency of treatment of locally advanced and metastatic NSCLC can be achieved in appropriately selected (mainly based on genetic predisposition) groups of patients in whom molecularly targeted drugs are used, e.g., erlotinib, gefitinib, and crizotynib [3, 4]. However, these drugs are reserved only for group of patients who have certain molecular abnormalities (depending on the race 9–51 % of patients with NSCLC have *EGFR* activating mutations; 3–7 % have *ALK* rearrangements) [5–7]. Therefore, still an overwhelming number of patients receive standard CTH. Recent research indicate that even in this group of patients, genetic predispositions can be used to select potentially the most effective treatment regimen, which will not only prolong the life of patients, but also improve the quality of life [8, 9].

Vinorelbine (5′-noranhydro vinblastine, VNR) is a semisynthetic derivative of vinblastine with antitumor activity. Like other *vinca* alkaloids (Lat. *Vinca rosea*, *Catharanthus roseus*), i.e., vinblastine and vincristine, this cytostatic has been applied, in the treatment of different cancers. Vinorelbine, like the other *vinca* alkaloids and taxanes, exerts its action by binding to tubulin. As a result of tubulin polymerization blocking (reverse mechanism than in the case of taxanes), depolymerization occurs followed inhibition of cells division at mitosis (phase-specific drug acting on the M phase of the cell cycle), which in turn leads to apoptosis of the cell [10]. As in the case of taxanes, in the mechanism of resistance to vinorelbine, certain disorders in functioning of the stathmin-1 protein may be critical (oncoprotein-18 and, stathmin-1 STMN1).

STMN1 is a protein whose expression is regulated by the product of *p53* suppressor gene. Experimental studies have shown that STMN1 significantly affects the dynamics of microtubules [11, 12]. To date, in available literature, there is only one publication that contains data on influence of any of the polymorphisms in the *STMN1* gene (including −2166T>C, rs182455) on the effectiveness of any of the currently used CTH regimens in lung cancer [13]. However, on the basis of information about the correlation between the expression of *STMN1* and efficacy of therapies based on the taxane or *vinca* alkaloids and that −2166T>C single nucleotide polymorphism (SNP) is located within or close to the putative transcriptional control region [14], it can be concluded that there exists potential predictive importance of this kind of genetic change (polymorphisms) in lung cancer patients.

The aim of this study was to determine the association between a SNP of *STMN1* gene promoter (−2166T>C) and the effectiveness of chemotherapy based on platinum compounds and vinorelbine in patients with unresectable, locally advanced or metastatic NSCLC.

## Materials and methods

### Study group

The study included 110 Caucasians patients with inoperable, locally advanced, and advanced NSCLC (IIIB and IV), treated in 2010–2013 at the Department of Pneumonology, Oncology and Allergology, Medical University of Lublin. In order to participate in the study, written informed consent for use of clinical data and peripheral blood were obtained from each patient. The study design was approved by the Committee of Ethics and Research at the Medical University of Lublin (consent no.: KE-0254/142/2010). NSCLC diagnoses were made on the basis of the result of histological or cytological examination of material obtained from primary tumor or metastases. Characteristics of the study group are presented in Table 1. All patients received the first line of CTH-standard doublet scheme based on platinum compound and vinorelbine. Disease severity at the start of treatment was assessed according to the TNM classification (VII edition by UICC). The median number of first-line CTH cycles was 4. In the study group, 30 % of patients were subjected to RCTH. Subsequent lines of therapy (using drugs such as erlotinib, docetaxel, or pemetrexed) were used for 28.2 % of patients. On the basis of the interview and the available medical records, detailed demographic and clinical data (gender, age, smoking status, histological type of tumor, stage of disease, weight loss, performance status (PS), presence of hematological disorders, the incidence of cancer in the family), and on the course of treatment (type and number of CTH or RCTH cycles, side effects and the effectiveness of therapy) were collected. Response to treatment was assessed by response evaluation criteria in solid tumors (RECIST, version 1.1). The occurrence of adverse events was assessed on the basis of the common toxicity criteria (CTC) guidelines (version no. 4.03). We also evaluated the differences in the length of progression-free survival (PFS) and OS in patients treated with first-line CTH depending on the clinical features and analyzed genetic polymorphism.

**Table 1:** Patient characteristics

Variable	Study group (*n* = 110)
Sex	
Male	85 (77.3 %)
Female	25 (22.7 %)
Age (years)	
Median	61
Mean ± std dev.	61.3 ± 8.9
Range	38–77
Smoking status (pack-years)	
Median	41.3
Mean ± std dev.	40 ± 23.1
Non-smokers	5 (4.5 %)
Current smokers	75 (68.2 %)
Former Smokers	27 (24.6 %)
No data	3 (2.7 %)
Histopathological diagnosis	
Adenocarcinoma	11 (10 %)
Squamous cell carcinoma	78 (70.9 %)
Large cell carcinoma	8 (7.3 %)
NOS (not otherwise specified)	13 (11.8 %)
Stage of disease	
IIIB	56 (50.9 %)
IV	54 (49.1 %)
Performance status	
PS = 0	30 (27.3 %)
PS ≥ 1	15 (72.7 %)
Weight loss before CTH	
Yes	67 (60.9 %)
No	33 (30 %)
No data	10 (9.1 %)
Anemia before CTH	
Yes	70 (63.6 %)
No	40 (36.4 %)
Number of cycles of first-line CTH	
Median	4
Mean ± std dev.	3.7 ± 1.5
Side effect after first-line CTH	
Yes	60 (54.5 %)
No	40 (36.4 %)
No data	10 (9.1 %)
Radiotherapy	
Yes	33 (30 %)
No	77 (70 %)
Subsequent lines of treatment	
Yes	79 (71.8 %)
No	31 (28.2 %)

### DNA extraction and genotyping

**Fig. 1:**
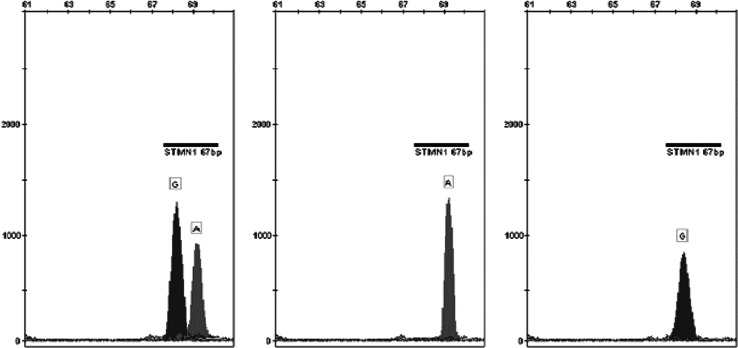
Example of genotyping results obtained by capillary electrophoresis of the SNaPshot PCR products. *From left* CT heterozygote, TT, and CC homozygote (analysis performed on the opposite strand where A=T and G=C)

For the isolation of DNA from peripheral blood leukocytes, a set of QIAamp DNA Blood Mini Kit (Qiagen, Canada) was used. Evaluation of the isolated DNA was made using a spectrophotometer BioPhotometer plus, cuvette equipped with filters UV/Vis (Eppendorf, Germany). *STMN1* gene SNP analysis was performed using a mini-sequencing technique (SNaPshot™ PCR). For the reaction, a set of ABI PRISM^®^ SNaPshot™ Multiplex Kit (Life Technologies, USA) was used (representative results of genotyping obtained by SNaPshot PCR products capillary electrophoresis is shown on Fig. 1).

### Statistical analysis

Genotyping results of analyzed gene were retrospectively associated with: objective response (OR) to treatment, the length of PFS, and OS of the patients. The statistical analysis of the results was performed using the computer software: Statistica 10 (Statsoft, USA), MedCalc 10 (MedCalc Software, Belgium). Results with the values of *p* < 0.05 were considered statistically significant. Using the Chi-square (*χ*^2^) test, the balance of the Hardy–Weinberg (HW) was rated, relationship of a series of demographic and clinical factors with the distribution of polymorphic variants of the *STMN1* gene and the impact of this SNP on OR. The Kaplan–Meier method was used to compare the probability of PFS and OS in patients with distinct clinical–demographic characteristics and the genotypes of the analyzed gene. Cox regression model with a stepwise selection with minimum AIC factor (Akaike Information Criterion) was used to assess which of the clinical and genetic factors affect survival. The Mann–Whitney *U* test was used to compare the median survival in the groups having various distributions of demographic, clinical, and genetic factors.

## Results

### Response to treatment

The distribution of *STMN1* genotypes (−2166T>C) did not depend on factors such as gender, age, histological subtype, stage of disease, PS, or smoking status (Table 2). Genotypes of *STMN1* gene were in Hardy–Weinberg equilibrium. CC, CT, and TT genotypes of *STMN1* gene (−2166T>C) occurred, respectively, in 45.4, 47.2 and 7.4 % of patients.

**Table 2:** *STMN1* gene genotype distribution according to demographic and clinical factors

Variable	*STMN1* (−2166T>C)			*p*, *χ* ^2^
	Genotype CC 49 (45.4 %)	Genotype CT 51 (47.2 %)	Genotype TT 8 (7.4 %)	
Sex				
Male	37 (44.1 %)	39 (46.4 %)	8 (9.5 %)	0.2891
Female	12 (50 %)	12 (50 %)	–	2.482
Age (years)				
<70	38 (46.4 %)	37 (45.1 %)	7 (8.5 %)	0.6141
≥70	11 (42.3 %)	14 (53.9 %)	1 (3.8 %)	0.975
Smoking status				
Current smokers	35 (47.9 %)	31 (42.5 %)	7 (9.6 %)	0.3915
Ex-smokers	11 (40.7 %)	15 (55.6 %)	1 (3.7 %)	4.109
Non-smokers	1 (20 %)	4 (80 %)	–	
Histopathological diagnosis				
Adenocarcinoma	5 (41.7 %)	6 (50 %)	1 (8.3 %)	
Squamous cell carcinoma	34 (44.7 %)	37 (48.7 %)	5 (6.6 %)	0.9300
Large cell carcinoma	5 (62.5 %)	2 (25 %)	1 (12.5 %)	1.885
NOS NSCLC	5 (41.7 %)	6 (50 %)	1 (8.3 %)	
Stage of disease				
IIIB	26 (48.15 %)	24 (44.45 %)	4 (7.4 %)	0.8252
IV	23 (%)	27 (%)	4 (%)	0.360
Performance status				
PS = 0	14 (46.7 %)	14 (46.7 %)	2 (6.6 %)	0.9759
PS ≥ 1	35 (44.9 %)	37 (47.4 %)	6 (7.7 %)	0.049

In the whole group, there were no patients with complete remission (CR) as a result of first-line CTH treatment. Control of the disease was observed in 59.1 % of patients, of which partial remission (PR) and stable disease (SD) occurred in 25.5 and 33.6 % of patients. Progression disease (PD) was observed in 40.9 % of patients. The incidence of PD was higher in patients with body weight loss compared with those with unaltered weight (*p* = 0.046, *χ*^2^ = 4). In the case of other demographic and clinical factors such as gender, age, smoking status, histological type of tumor, PS, stage of disease, the use of RCTH, or the incidents of cancer in the family, there were no statistically significant differences in the possibility of obtaining response to first-line CTH treatment. Moreover, SNP (−2166T>C) of *STMN1* gene did not affect significantly the type of response to first-line treatment.

### Progression-free survival

**Fig. 2:**
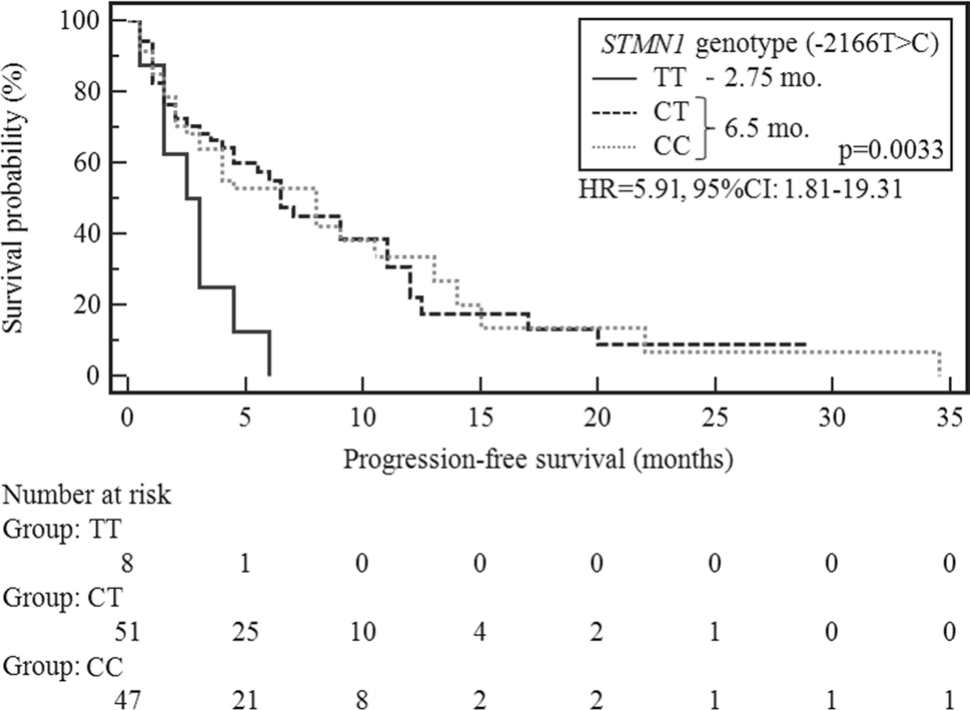
Probability of progression-free survival change depending on *STMN1* genotype

In the study population, median PFS (mPFS) was 6.5 months. In patients who had no weight loss before CTH, mPFS was significantly longer compared with other patients (8 vs. 3 months; *p* = 0.0063, *χ*^2^ = 7.46; HR 0.47, 95 % CI 0.27–0.81). In patients without anemia before CTH, mPFS were significantly longer compared with those who had this type of hematological disorders (8 vs. 4.5 months; *p* = 0.011, *χ*^2^ = 6.46; HR 0.54, 95 % CI 0.33–0.87). Patients undergoing RCTH characterized by significantly longer mPFS compared with those treated only CTH (9 vs. 4 months; *p* = 0.0247, *χ*^2^ = 5.04; HR 0.58, 95 % CI 0.36–0.93). Patients with no cases of cancer in the family had a significantly longer mPFS compared WITH other patients (6 vs. 1.5 months; *p* = 0.0168, *χ*^2^ = 5.72; HR 0.28, 95 % CI 0.10–0.79). In addition, carriers of the TT genotype (−2166T>C) of *STMN1* gene demonstrated significant shortening in mPFS compared with patients with other polymorphic variants of this gene (2.75 vs. 6.5 months; *p* = 0.0033, *χ*^2^ = 8.63; HR 5.91, 95 % CI 1.81–19.33; Fig. 2). In the case of other demographic and clinical factors, there were no statistically significant differences in the duration of PFS in the study group.

Cox multivariate logistic regression demonstrated that the factors that significantly shortened PFS in a group of patients treated with CTH based on platinum compounds and vinorelbine (overall fit of the model: *p* = 0.0021, *χ*^2^ = 47.28) were: poor PS (*p* = 0.0008; HR 5.24, 95 % CI 1.99–13.79) and diagnosis of non-squamous type of NSCLC (*p* = 0.0188; HR 3.28, 95 % CI 1.22–8.78).

### Overall survival

**Fig. 3:**
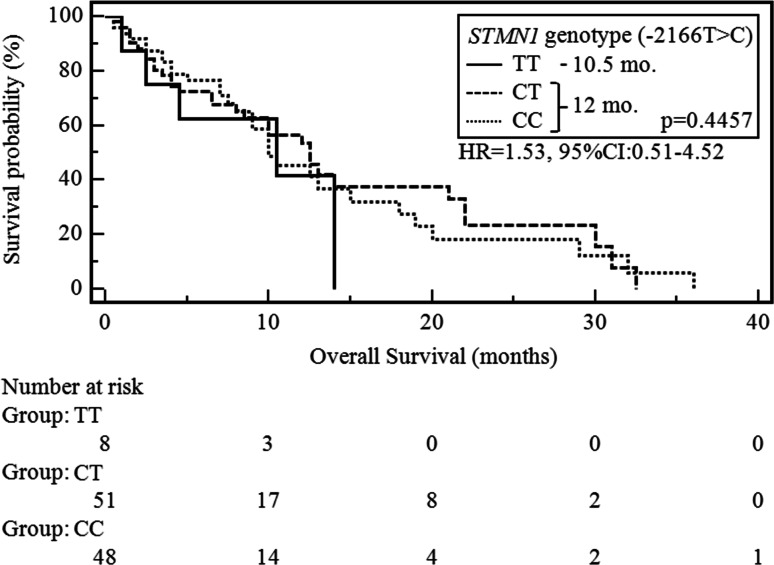
Probability of overall survival change depending on *STMN1* genotype

In the study population, median OS (mOS) was 12 months. Patients in good PS had significantly longer mOS compared with other patients (19 vs. 10 months; *p* < 0.0392, *χ*^2^ = 4.25; HR 0.58, 95 % CI 0.35–0.97). In patients who had no weight loss before CTH, mOS was significantly longer compared with other patients (14 vs. 9 months; *p* = 0.0016, *χ*^2^ = 9.91; HR 0.39, 95 % CI 0.21–0.70). In patients without anemia before CTH, mOS was significantly longer compared with those who had this type of hematological disorders (15 vs. 10 months; *p* = 0.0362, *χ*^2^ = 4.38; HR 0.59, 95 % CI 0.36–0.97). Patients with no cases of cancer in the family had a significantly longer mOS in comparison with other patients (13 vs. 8 months; *p* = 0.0134, *χ*^2^ = 6.11; HR 0.27, 95 % CI 0.09–0.76). In the case of other demographic and clinical factors, there were no statistically significant differences in the duration of OS in the study group. There was no significant association between mOS and the occurrence of particular *STMN1* genotypes (Fig. 3).

Cox multivariate logistic regression demonstrated, that the factor, that significantly shortened OS in the study group (overall model fit: *χ*^2^ = 55.05, *p* = 0.0001) was only poor PS (*p* < 0.0001; HR 30.77, 95 % CI 7.93–119.36).

All results of response to treatment, PFS, and OS are presented in Table 3.

**Table 3:** Effect of demographic, clinical, and genetic factors on: response rates, progression-free survival, and overall survival in the study group

Variable	PD 45 (40.9 %)	SD, PR 65 (59.1 %)	*p*, *χ* ^2^	*p*, OR 95 % CI	Median PFS (months)	*p*, *χ* ^2^	HR (95 % CI)	Median OS (months)	*p*, *χ* ^2^	HR (95 % CI)
Sex				0.5707						
Male	36 (42.4 %)	49 (57.6 %)	0.7365	0.7656	6	0.9802	0.9931	12	0.9310	1.0260
Female	9 (36 %)	16 (64 %)	0.113	0.3042–1.9271	6.5	0.0006	(0.5751–1.7148)	12.5	0.0075	(0.5746–1.8320)
Age (years)				0.2828						
≤70	32 (38.1 %)	52 (61.9 %)	0.3950	1.6250	6.5	0.8690	1.0486	12	0.9612	0.9851
>70	13 (50 %)	13 (50 %)	0.724	0.6700–3.9412	6	0.0272	(0.5965–1.8435)	14	0.0024	(0.5373–1.8059)
Smoking status				0.3462						
Smokers	43 (42.2 %)	59 (57.8 %)	0.6047	0.3430	6	0.6125	0.7620	12	0.3916	0.6194
Non-smokers	1 (20 %)	4 (80 %)	0.268	0.037–3.1783	12	0.2566	(0.2662–2.1814)	20	0.7339	0.2070–1.8532)
Histopathological diagnosis										
Adenocarcinoma	6 (54.5 %)	5 (45.5 %)	0.2632	–	9	0.4678	–	8.5	0.3924	–
Squamous cell carcinoma	28 (35.9 %)	50 (64.1 %)	3.984		6.5	2.5418		12.5	2.9948	
Large cell carcinoma	3 (37.5 %)	5 (62.5 %)			5			10		
NOS NSCLC	8 (61.5 %)	5 (38.5 %)			3			7		
Stage of disease				0.1310						
IIIB	19 (33.9 %)	37 (66.1 %)	0.1860	1.8083	8	0.4733	0.8382	12.5	0.6224	0.8838
IV	26 (48.1 %)	28 (51.9 %)	1.749	0.8383–3.9005	4	0.5142	(0.5174–1.3580)	10	0.2424	(0.5404–1.4453)
Performance status				0.0668						
PS = 0	8 (26.7 %)	22 (73.3 %)	0.1004	2.3663	8	0.1105	0.6675	19	0.0392*	0.5810
PS ≥ 1	37 (46.3 %)	43 (53.7 %)	2.699	0.9422–5.9428	5.5	2.5464	(0.4063–1.0967)	10	4.2542	(0.3468–0.9734)
Weight loss before CTH				0.0287						
Yes	19 (57.6 %)	14 (42.4 %)	0.0456*	0.3852	8	0.0063*	0.4672	14	0.0016*	0.3879
No	23 (34.3 %)	44 (65.7 %)	3.997	0.1638–0.9056	3	7.4580	(0.2706–0.8067)	9	9.9083	(0.2151–0.6996)
Anemia before CTH				0.5828						
Yes	30 (42.9 %)	40 (57.1 %)	0.7277	0.8000	4.5	0.0110*	0.5395	10	0.0362*	0.5865
No	15 (37.5 %)	25 (62.5 %)	0.121	0.3608–1.7736	8	6.4581	(0.3352–0.8684)	15	4.3857	(0.3560–0.9664)
Radiotherapy				0.0602						
Yes	9 (27.3 %)	24 (72.7 %)	0.0905	2.3415	9	0.0247*	0.5785	13	0.0523	0.6093
No	36 (46.7 %)	41 (53.3 %)	2.865	0.9641–5.6865	4	5.0444	(0.3588–0.9327)	10	3.7663	(0.3694–1.0049)
Side effect after CTH										
Yes	–	–	–	–	6.5	0.3239	0.7772	12.5	0.8243	1.0634
No					4	0.9730	(0.4710–1.2824)	10.5	0.0493	(0.6180–1.8300)
Subsequent lines of treatment										
Yes	–	–	–	–	7	0.7040	0.9099	14	0.2181	1.3793
No					6	0.1444	(0.5591–1.4809)	10	1.5169	(0.8268–2.3012)
Family history of cancer (any malignant)				0.2156						
Yes	6 (60 %)	4 (40 %)	0.3660	0.4103	1.5	0.0168*	0.2783	8	0.0134*	0.2690
No	16 (38.1 %)	26 (61.9 %)	0.817	0.1001–1.6806	6	5.7182	(0.09756–0.7940)	13	6.1112	(0.09498–0.7619)
*STMN1* (−2166T>C)										
CC	19 (38.8 %)	30 (61.2 %)	0.1386	–	8	0.0196*	–	10	0.7296	–
CT	20 (39.2 %)	31 (60.8 %)	3.952		6.5	7.8605		12.5	0.6306	
TT	6 (75 %)	2 (25 %)			2.75			10.5		
*STMN1* (−2166T>C)				0.5788						
CC	19 (38.8 %)	30 (61.2 %)	0.7193	1.2440	8	0.5622	0.8697	10	0.9090	1.0296
CT lub TT	26 (44 %)	33 (66 %)	0.129	0.5754–2.6894	6	0.3360	(0.5425–1.3942)	12.5	0.0131	(0.6248–1.6965)
*STMN1* (−2166T>C)				0.6252						
CT	20 (39.2 %)	31 (60.8 %)	0.7694	1.2109	6.5	0.5559	0.8682	12.5	0.6437	0.8894
CC lub TT	25 (43.8 %)	32 (56.2 %)	0.086	0.5618–2.6099	4.5	0.3469	(0.5424–1.3896)	10.5	0.2139	(0.5412–1.4617)
*STMN1* (−2166T>C)				0.0663						
TT	6 (75 %)	2 (25 %)	0.1064	0.2131	2.75	0.0033*	5.9067	10.5	0.4457	1.5260
CC lub CT	39 (39 %)	61 (61 %)	2.607	0.0409–1.1097	6.5	8.6347	(1.8066–19.313)	12	0.5815	(0.5150–4.5221)

## Discussion

Selecting the most appropriate cytostatics combination is based primarily on the patients’ PS, adverse events, convenience of use, experiences, and personal preferences of physician. However, each of the available CTH regimens, regardless of the treatment line, is characterized by significant differences in efficiency, i.e., in response rates, duration of PFS, and OS observed in individual patients. It is obvious that the resistance to systemic treatment does not apply equally all the patients, and is likely due to molecular differences.

Changes in the structure, expression, or function of various proteins may depend on the occurrence of SNPs in coding or noncoding sequences (mainly promoter) of different genes [15]. Molecular changes with potentially highest impact on the effectiveness of CTH are mainly related to the metabolism and mechanism of action of the chemotherapeutic agent. In contrast to the expression determined in tumor tissue, SNPs analysis can be carried out in materials that are easy to obtain (e.g., DNA from peripheral blood leukocytes). Therefore, many studies (unfortunately mostly retrospective) examined the effects of the different polymorphic variants of various genes on the effectiveness of different treatment regimens [16–18].

In our recently published study, we have shown that SNP −2166C>T can be considered as a biomarker of the treatment efficiency in advanced NSCLC patients treated in second line of chemotherapy (monotherapy) with use of docetaxel or paclitaxel. These drugs are affecting mitotic spindle (similarly to vinorelbine which is currently used in first-line chemotherapy) [13]. In the available literature, there are no more publications containing data on the role of any *STMN1* gene polymorphisms (including −2166C>T) as risk, prognostic, or predictive factor in NSCLC patients. However, on the basis on the relationship of changes in *STMN1* gene expression and efficacy of therapies based on the taxane or *vinca* alkaloids in cell cultures and in cancer patients, it can be concluded about the potential importance of such genetic change. Despite the fact that the actual functional relevance of STMN1 polymorphisms on the level of gene expression and protein function has not yet been established, SNPs in regulatory regions frequently affect gene expression. SNPs in the STMN1 gene alter the recognition motif and thus the potential binding of a transcription factor. Studied SNP is localized next to a consensus sequence of a glucocorticoid responsive element (steroidal hormones have been shown to modulate STMN1 expression and protein function) [14]. In studies of Alli et al. and Balachandran et al. [11, 12] carried on breast and ovarian cancer cell lines, it was demonstrated that the increased expression of STMN1 had reduced microtubule polymerization (which significantly reduced binding of paclitaxel). Some studies also suggest that high expression of STMN1 may be associated with resistance not only to the taxanes but also to *vinca* alkaloids [19, 20]. Increased expression of this protein seems to have no influence on the effect of drugs, in which mechanism of action is not related with formation of microtubules. In a study of 54 female patients with advanced breast cancer who have received docetaxel as neoadjuvant CTH, Meng et al. [21] showed that low level of STMN1 expression is a good predictor indicating a high probability of response to applied therapy.

Moreover, to date only two studies assessed the association between the mRNA expression of *STMN1* gene and disease progression or OS duration in patients treated with CTH. Among these publications, only one study concerned the NSCLC patients (612 patients treated with CTH based on a combination of platinum with gemcitabine or vinorelbine, tissue material was available only from 75 patients). In the subgroup of patients treated with the combination of platinum compounds and vinorelbine, statistically significant effect of *STMN1* gene expression (expression level ≤ 5 vs. > 5) on PFS duration (6.4 vs. 2.1 months, *p* = 0.05) has been shown. However, a significant relationship between *STMN1* gene expression and OS has not been reached. In quoted publication, the threshold cycle (CT) was the fractional cycle number at which the fluorescence generated by cleavage of the probe exceeded a fixed level above baseline. The relative amount of tissue target mRNA standardized against the amount of β-actin mRNA was expressed as $-\Delta {\text{CT}} = \left[ {{\text{CT}}_{{\left( {{\text{target gene - }}1} \right) }} - {\text{CT}}_{{(\upbeta - {\text{actin}})}} } \right]$. The ratio of the number of target mRNA copies to the number of b-actin mRNA copies was then calculated as $2^{{ -\Delta {\text{CT}}}} \times K$, where *K* is a constant [20]. In the next study, Jilăveanu et al. evaluated the relationship between expressions of different proteins (including STMN1) with duration of PFS. Biomarker rating was carried out in 263 melanoma patients treated with the combination of carboplatin, paclitaxel, and sorafenib. However, no significant association between STMN1 expression and duration of PFS and OS was observed [22].

In this study, we have shown for the first time that TT genotype of STMN1 gene (−2166T>C) may be associated with shortening of PFS in patients with locally advanced or metastatic NSCLC who received first-line chemotherapy based on a combination of platinum compounds and vinorelbine. Limitation of our study was heterogeneous population (part of patients were subjected to chemoradiotherapy and/or subsequent lines of CTH).

## Conclusion

Rare TT genotype of *STMN1* gene may be an unfavorable predictive factor of chemotherapy based on platinum compounds and vinorelbine, in patients with NSCLC.
